# Resilience to Leaking — Dynamic Systems Modeling of Information Security

**DOI:** 10.1371/journal.pone.0049804

**Published:** 2012-12-05

**Authors:** Kay Hamacher

**Affiliations:** Department of Physics, Department of Computer Science, and Department of Biology, Technische Universität Darmstadt, Darmstadt, Germany; UMIT, Austria

## Abstract

Leaking of confidential material is a major threat to information security within organizations and to society as a whole. This insight has gained traction in the political realm since the activities of Wikileaks, which hopes to attack ‘unjust’ systems or ‘conspiracies’. Eventually, such threats to information security rely on a biologistic argument on the benefits and drawbacks that uncontrolled leaking might pose for ‘just’ and ‘unjust’ entities. Such biological metaphors are almost exclusively based on the economic advantage of participants. Here, I introduce a mathematical model of the complex dynamics implied by leaking. The complex interactions of adversaries are modeled by coupled logistic equations including network effects of econo-communication networks. The modeling shows, that there might arise situations where the leaking envisioned and encouraged by Wikileaks and the like can strengthen the defending entity (the ‘conspiracy’). In particular, the *only* severe impact leaking can have on an organization seems to originate in the exploitation of leaks by another entity the organization competes with. Therefore, the model suggests that leaks can be used as a `tactical mean’ in direct adversary relations, but do not necessarily increase public benefit and societal immunization to ‘conspiracies’. Furthermore, within the model the exploitation of the (open) competition between entities seems to be a more promising approach to control malicious organizations : *divide-et-impera* policies triumph here.

## Introduction

Information and communication systems are connected – technically, legally, economically, and socially – to the outside world. The integrity and confidentiality of the information contained therein can be under attack from out- and inside attackers. Typically, technical measures can assure to some extent the security against *outside* attacks by, e.g., general cryptographic protocols [1] and tailor-made protocols for particular application, e.g., for privacy in medicine [2], [3]. Operational security [4], [5] on the one hand and threats by insiders [6], [7] on the other hand remain the main source of concern.

### Previous Work on Insider Threats

Previous work on insider threats focused on identification [8]–[11], behavioral effects [6], [12], [13], particular areas of threat mitigation [14], or interfering with malicious behavior [15].

In contrast to this previous work, this study models the overall effects of insider activities, such as intentional leaking of confidential information. The most prominent activity related to intentional leaking of sensitive information was started by the Wikileaks platform. Here, we will not focus on its leaks, the intentions of sources, or the actors behind Wikileaks. We will, however, use the stated intentions of Wikileaks’ supporters and founders as a guideline for our analysis. We propose to revise the employed simple, linear, direct cause-and-effect picture. As it neglects both, economic insight and knowledge on systems theory.

This work is built on large-scale simulations of the modeled society and the information systems immersed in this society. Previous work showed that for the investigation of non-trivial effects in complex security settings only such simulations are capable of generating the necessary details, e.g., Hamacher and Katzenbeisser [16] were able to refute conventional wisdom like the “more data is better” paradigm for telecommunication data retention. The complex nature of communication behavior and the implied pattern and outlier recognition problem could only be analyzed via such computational procedures. Furthermore, Bonabeau [17], [18] has extensively discussed the necessity of such simulations for organizatorial and operational risk in financial firms, for which information security is of paramount importance.

### Wikileaks’ Underlying Idea

Although we will not restrict our analysis on Wikileaks and its implications alone, we nevertheless will use the underlying idea of indiscriminate leaking of information as a basal model for threat and attacks on information and communication systems. The ‘founder’ of Wikileaks, Julian Assange posted on his former website http://iq.org two self-published papers on the underlying philosophy. These documents are still available under [19].

The key quote is of special importance for our economic and dynamical system analysis on the information security of systems threatened by Wikileaks’ style of attacks:

*“The more secretive or unjust an organization is, the more leaks induce fear and paranoia in its leadership and planning coterie. This must result in minimization of efficient internal communications mechanisms (an increase in cognitive “secrecy tax”) and consequent system-wide cognitive decline resulting in decreased ability to hold onto power as the environment demands adaption.”*

Now, this thinking constitutes biologism, which is the school of thought that tries to explain social behavior by biological principles. This notion is easily identifable in Assange’s texts: the ‘decreased ability’ is due to the fitness (dis)advantage of an entity, while ‘the next action’ refers to the fact, that the dynamics effectively forms a Markov chain. The overall idea is thus the “throttling” of “conspiracies” by reducing link weights and *not* eliminating individual nodes.

To put this notion into a more abstract and general framework: the more the internal communication of an entity relies on secrecy, the more severely the sustainability of the organization is reduced by information leaks. Note, that such an entity is not necessarily a real ‘conspiracy’, but in the Wikileaks-ideology rather any formal or informal collection of actors – from states & governments, over companies, to informal groups such as illegal monopolies or criminal syndicates. At this point, Assange’s biologism and real economic thinking converge.

However, the devil is in the details, as almost always in economics. In particular, it is in no way obvious whether the real impact of leaking is substantial and how feedback and competition among various actors influence the outcome. To understand such potentially non-linear effects, one needs to model the *dynamics* of leaking effects (including potential feedback mechanism). A promising route to this end is dynamic systems theory [20].

## Materials and Methods

In this section, we will give a step-by-step justification of our model for the dynamics of leaking and economic ongoings within the framework of dynamical systems modeling (see the final model of Eqs. 2 in Section “Modeling Leaking Dynamics”.

We start from a simple model of open and socially acceptable competition and resource constraints. We then proceed to include effects of leaks on the organizations’ performance and viability.


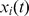
 will be the (relative) size of an entity *i* using an internal information or communication system at time *t*. In reference to the Wikileaks philosophy (see Sec. “Wikileaks’ Underlying Idea”) the 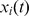 is the fraction of actors participating in a ‘conspiracy’.

A well established model for the growth of an economically active entity is the logistic map [21]–[23]

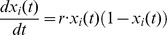
that was shown to produce non-trivial, complex dynamics [24]–[26]. Here, *r* is the growth rate. We assume 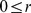 to ensure the tendency to grow, whenever the entity *i* exists. Whenever the environment is unfavorable for *i*, then the growth rate might become negative (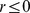). Now, *r* is constant in trivial logistic map models. In the subsequent parts of this section we will, however, develop a functional form, so that it depends on several effects (most prominently the implications of leaking). Note, that 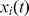 is not an absolute number of entity members, but rather a ratio.

Resource constraints and feedback loops are the most important boundary conditions for the dynamics. Among others, such constraints are:

- A ‘conspiracy’ can necessarily only be a (tiny) fraction of the overall population. Thus, there exists an upper bound on the number of ‘conspirators’; in a more neutral formulation, only a fraction in a society needs to be considered, otherwise we would face a monopolistic situation which follows completely different rules.
- Coordination Problems: a ‘secret’ group cannot rely on official enforcement schemes of contracts (law, legal codes, judges, 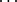), thus it needs either to establish mechanisms on its own (covering the inherent costs) or its size is bounded to ensure direct & personal interactions, only.
- Cognitive/Social/Trust Resource: in a ‘secret’, unofficial group ‘contracts’ cannot of officially be enforced, thus trust and reputation are the most important mechanism. However, number of people to whom one can maintain reliable social relations is bounded by Dunbar’s number [27] of some 150 persons.

All of the above leads to a saturation value for each 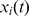 at all times *t*. Eventually, this value is the so-called carrying capacity *K* of the society, which we assume to be homogenous for all *i*. We thus arrive at the logistic equations including a carrying capacity:
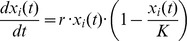



We discuss the choice of the unknown parameter *K* later.

### Competition Among Entities

In reality entities interact. Such interactions can be beneficial or disadvantageous for an entity, e.g., when there exists competition for shared, but limited resources. Such models have a direct companion in biology in the notion of mutualism [28].

These effects are modeled on the basis of *coupled* logistic equations:
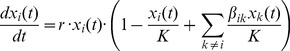
(1)


Note, that we model only instantaneous competition and neglect retardation and memory effects. This relates to the biologistic assumption mentioned in the Introduction: an entity ‘computes’ it next action on the basis of the last outcome and event – thus a Markov chain. The Markov property is in close analogy to the nowadays disputed rational model of market participant.

The parameters 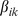 quantify the strength of interaction. They are positive for mutually supporting or synergistic interactions of entity *k* with entity *i*; 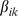 is negative if *k* and *i* are in an overall competitive setting, where a larger size of *k* implies, e.g., less resources for *i* and thus reduced or even inverted growth (reduction). Note, that generally the situation can be asymmetric 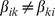.

### Modeling Leaking Dynamics

Leaking of internal information of an entity and thus breach of information or communication security can have effects on several levels. To include these effects we extend Eq. 1 to the following coupled logistic equations:
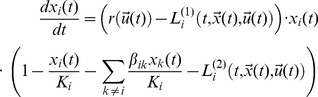
(2)


Here, 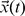 and 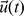 are *n*-dimensional vectors with entries 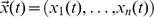 and 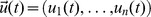, respectively.

The effects of leaking are modeled by the terms 
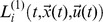
 and 
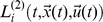
. These leaking terms 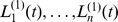 and 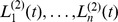 depend on the abilities of the ‘conspiracies’, thus on the 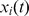 and the utilities 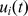 of the (in)formal information and communication networks they are comprised of. Each individual utility 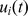 in turn depends on the value 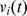 an information or communication network has for the respective entity *i*. We employ Bernoulli’s cardinal utility 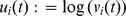 to account for the diminishing marginal utility of wealth or value.

We set the *effective* growth rate *r* of Eqs. 2 to the *economic value* of the entities 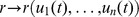 and introduce 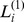 to account for several effects, that are related to the allocation of resources and thus the economic value of the information systems/networks the entity can command over:

1. the “attractiveness” to join that entity as, e.g., an employee or a ‘co-conspirator’.
2. the resources an entity can invest in growth. The more pronounced the differences to other entities competing with it, the better a particular entity will perform, thus grow.
3. Additionally, leaking is more likely the larger the entity as there is just more data to be leaked. Also leaking is more likely, whenever the society via its social norms accepts leaking.
4. we need to include econo-behavioral effects in the growth rate, too. E.g., the more “powerful” an entity appears – thanks to leaking – the more likely voluntary joining by outsiders is.

A final effect of leaking 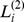 is the effect on entity has on the carrying capacity of another entity, e.g., the more powerful *j* the more it can use blackmailing to reduce carrying capacity for *i*. This effect, however, does not affect the actual growth rate, but the carrying capacity.

As a final step, we need to quantify the *economic value* of a network describing an entity and thus its ability to allocate and use resources.

### Resources of a Network – Economic Models

To fully address the economical (dis)advantages any entity faces through leaking, we need to include the *economic value* of the communication and information network via a model. In the literature, three models for the value 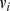 for a network of 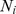 actors/nodes forming an entity *i* are predominant:

- Metcalfe’s law [29] 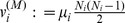

the value is proportional to the number of possible links between the 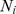 participants

- Reed’s law [30] 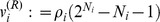

Here, the value is proportional to the number of all possible sub-groups that can be formed by the 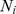 members of an entity

- Beckström [31]

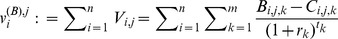



here, the overall value is the sum of interest 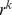 deflated values of all transactions *k* between participants *i* and *j* with benefits 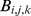 and costs 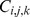 that occurred at time 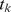.

In the following, we will restrict ourselves to Metcalfe’s law and Reed’s law as the much broader formulation by Beckström includes too many free parameters to sample those meaningfully; at the same time, Metcalfe’s law is a special case of Beckström’s one, thus we cover its implications in a simplified way.

## Results

### Focusing On Generic Set-Up

We decided to model the most generic situation with two competing entities immersed in a society (resembled by 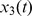). Thus 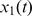 and 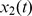 are our simulation variables, while 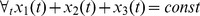 holds always. Therefore, any individual can only belong to one of the two entities 1 and 2 or to the rest of society.

Following the arguments in Section “Modeling Leaking Dynamics” we model the growth rate to be the larger the greater the differences between the utility 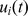 of the communication and information network of an entity *i* with an (in)formal value of 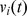. Thus, we set 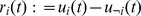, where 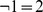 and 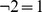. As there is nothing special about entity 1 and 2 the symmetric usage of 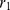 and 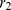 is justified without loss of generality.

For this basic set-up of two entities 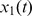 and 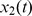 in a society 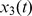 we set
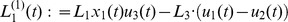



The rationale is a follows.

- first term: the larger entity 1, the higher the leaking probability while also the economic power of the society can encourage leaking as a potential whistle-blower can assume to find alternative employment opportunities;
- second term: the greater the econ. differences between the two entities, the larger the psychological effect/the motivation to join the more powerful one.

Note, that leaking and whistle-blowing are not the same concept. Rather, whistle-blowing is a special case of leaking with an ethical-moral motivation, while leaking can also occur for malicious or questionable reasons.

Furthermore, we set
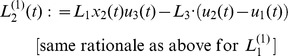


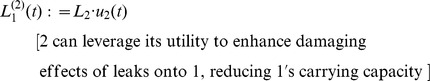


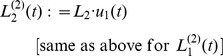


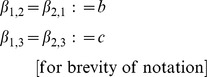
in the Eqs. 2, where 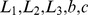 are the free parameters of this final model. Note, that the terms 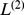 are strictly positive and thus reduce the carrying capacity.

We simulated and analyzed for Reed’s network value model and for Metcalfe’s model 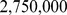 replicas each. We coped with the unknown parameters 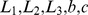 in the model by a randomized sampling over a parameter hyper-cube, which was manually optimized to cover the region of convergence and numerical stability. Thus, we effectively used a uniform prior on model parameters.

Furthermore, we set the carrying capacity to 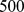 to be well above Dunbar’s number (see Sec. “Materials and Methods”). We integrated the resulting, non-linear ordinary differential equations numerically by the well-known Runge-Kutta algorithm [20], [32]. We obtained the full time-courses of all set-ups for times 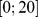 with time-steps of 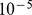 in arbitrary time units. We ensured that all simulations have converged to a steady state at 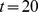 the latest.

## Results

We applied to each of the high-dimensional time series from the numerical integration of Eq. 2 dimensionality reduction via Principal Component Analysis (PCA) [33]: to this end, we extended the description vector 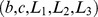 of one particular simulation by a binary variable (yes/no: did at least one entity 1 or 2 vanished) and an ‘asymmetry’ parameter to account for the relative differences in the sizes of 1 and 2 at the start of the simulation at 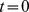 (to account for ‘unfairness’ at start).

We then processed these vectors further: we computed the 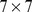 covariance-matrix of all these vectors. If there are any dominant influences of any of the parameters 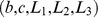 on the outcome (vanishing or not) present, then this would be detectable in the covariance matrix.

**Figure 1:**
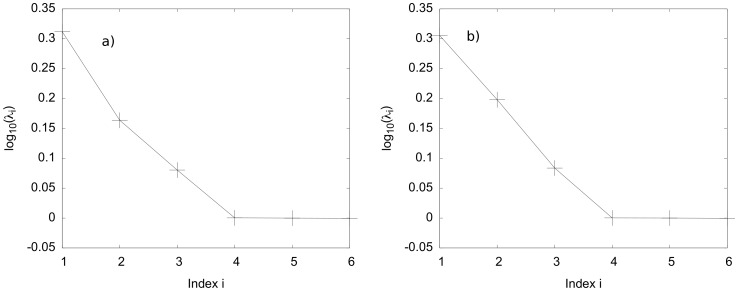
The eigenvalue spectrum for the PCA of the results in the (a) Metcalfe model – clearly, an exponential decay of the eigenvalues is visible, we fitted a linear model to the relevant four leading eigenvalues in the lin(*x*)-log(*y*)-data and found this statistical significant with a *p*-value of 0.01. (**b**) the same as in (a), here the linear model fit had a *p*-value of 0.002. (lines are guides to the eye).

**Figure 2:**
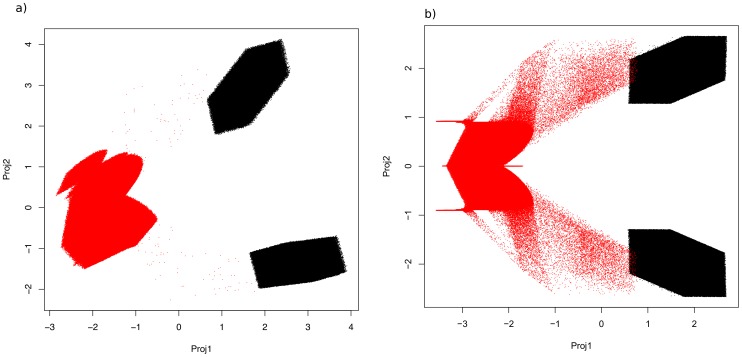
The projection of the respective simulation data using the two leading eigenvectors from Fig. 0 from the PCA for Metcalfe’s model (a) and (b) the same as in (c) for Reed’s model [black: one entity got extinct at some time 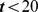, red: both entities survived until 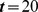.].

In Figures 1 and 2 we show our results for both economic models of network value. In the Figs. 1 a) and 1 b) we show the eigenvalue spectra obtained from the PCA procedure, which – due to the exponential decrease of eigenvalues – support the applicability of the PCA procedure for our data. Typically, one can reconstruct the original covariance matrix from all eigenvalues and -vectors. If, however, the major contributions stem from a low-dimensional manifold, then only some few eigenvalues and their corresponding vectors are necessary to this end. Figure 1 shows the entries in the eigenvectors of the leading eigenvalues in the PCA procedure.

**Figure 3:**
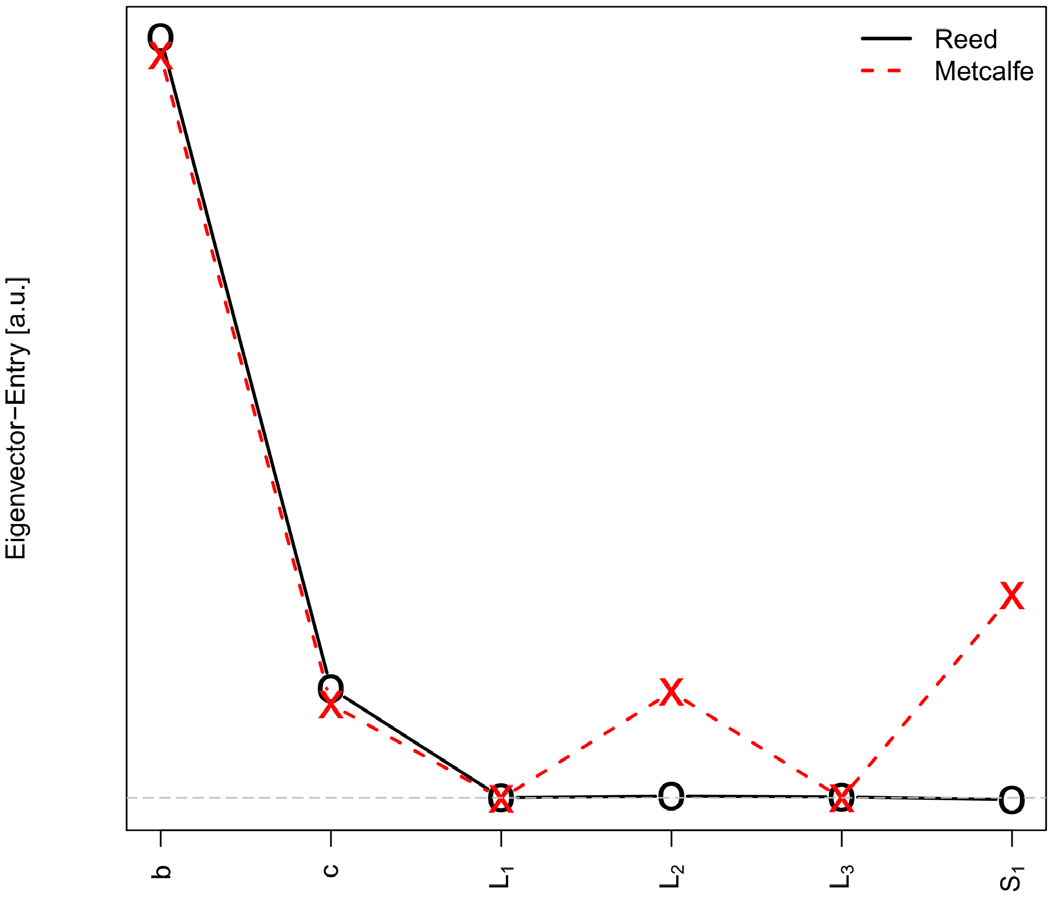
For both economic models (Metcalfe vs. Reed): (squared) entries of the eigenvectors for the leading eigenvalue of the PCA procedure for our simulation data. The black, horizontal, broken line indicates vanishing entries. 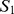 is proportional to the asymmetry of the starting sizes of the entities 1 and 2 at 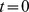.

It is obvious, that the separation of situations, in which one entity (either 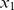 or 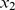) vanished, can be clustered within the data via the PCA analysis. This indicated that there exist subspaces of parameters 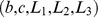 where one of the two scenarios occur. To identify, what the key driver of this behavior is, the leading eigenvector from the PCA was used. Figure 3 shows the absolute values of the eigenvector entries for the leading eigenvalue in the PCA.

We deduce from Figure 3 that in the Reed network value model, leaking is *not* responsible for the extinction of a group at all : the entries in the eigenvector for the terms modeling the leaking 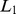, 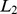, and 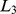 vanish all together and thus do not have any influence on the summarizing covariance matrix. Rather, competition among the entities (parameter *b*) is the key driver. To a smaller extent the competition or support with or from the society (parameter *c*) without leaking is responsible for extinction.

Leaking has only a minor effect in the Metcalfe network model, but to the same extent as the “fairness” of the starting situation (indicated by 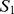). Thus leaking has as much impact as an unfavorable starting situation. And this leaking effect is only due to the process quantified by 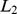. This is (see above, Section “Modeling Leaking Dynamics”) the ability of one entity to reduce the effective carrying capacity of the other entity by, e.g., lobbying or blackmailing with leaked information. The effects of competition and societal support (*b* and *c*) are the same as in Reed’s model.

Our results suggest furthermore, what happens to the remaining entity, if the competitor got extinct. In particular, the extinction of one entity lead almost always to an increase in the relative size of the stable and still existing one. We found for our two economic models for network value:

- Reed: in 59% of the simulations, one group got extinct, the remaining one grew to an average relative size of 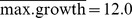 of its starting value at 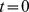

- Metcalfe: in 41% of the cases one group got extinct, the remaining entity grew to an average relative size of 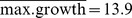

Non surprisingly, we can conclude, that the surviving entity is growing on the resources freed by the extinct competitor.

## Discussion

### Summary

In this paper we have addressed the impact of leaking of private, sensitive information of entities in a ‘Wikileaks-like’ scenario. We have motivated a quantitative feedback-model that builds upon economic models for information, social, and communication networks. The model includes several terms to account for leaking attacks on the long-term sustainability of an organization.

We found the overall effect of leaking proposed by Julian Assange for direct cause-and-effect situations to be seriously reduced or non-existent within our model. The main reason for the shortcomings of the simple biologistic picture in the ‘Wikileaks-attack mode’ is the feedback via competition and mutual support of entities that in the Wikileaks language are considered ‘conspiracies’.

In particular, we found competition between entities for the extinction of *one* ‘conspiracy’ or entity to be much more important than any other externality or leaking attack. Such a ‘ranking’ of influences is hardly possible in simple cause-and-effect thinking – only (semi-)quantitative understanding can provide such insight. Within our framework, the minor effect leaking can have is its usage as a tactical weapon of one entity to attack a competitor – thus, evaluation of the security impact of leaks need to take into account the ecosystem of competitors and their potential involvement. Also our models suggest that neither social norms and psychological effects (such as perception of participants and thus econo-behavioral effects) might have any noticeable effect in reality.

However, if one identifies our entities with ‘conspiracies’ then the extinction of one entity is almost always connected to opportunity costs: the super-proportional growth of the remaining entity. Thus fighting ‘conspiracies’ in this framework is always costly.

A *divide-et-impera* approach, that effectively controls both entities via their mutual competition turn s out to be more efficient as the combined influence of two existing entities can be smaller – at least within our model.

Thus we suggest as a hypothesis for future research and application that not only technical means of (IT-)security and operational security can be beneficial for *organizations* and the surrounding society. Rather, maintenance of a subtle balance of competition among adversaries might be a viable *public* security policy.

Why the Metcalfe scenario shows residual susceptibility towards 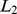-type leaking (leveraging one own utility/size to reduce carrying capacity of the opposing conspiracy) remains an open question and cannot be answered with the present study. It is suggestive to speculate about the importance of ‘cliques’ which are incorporated into Reed’s model: probably, the inclusion of cliques effects stabilizes against blackmailing. As such an effect would be included, however, only implicitly the modeling approach undertaken here is not amenable for such an elaborated analysis.

### Outlook

Beyond the above given hypothesis, there is more work to be done on the model itself, e.g., Extending the framework by several aspects might be desirable. However, one must be aware that this introduces more free parameters which renders a comprehensive study of all potential scenarios and configurations exponentially expensive. Nevertheless, we would like to give an incomplete list of such aspects worthwhile to consider in a future study. Among these are.

- *time-dependent* externalities & mutually dependent fitness functions(coevolution)
- stochastic fluctuations, via *stochastic* differential equations (SDEs), as is common in, e.g., dynamical system theory [34]
- more involved counter-strategies, e.g., exploiting leaks

In this study we refrained from introducing these aspects to focus on the most basic setting (consisting of two entities, competition, interaction with society, economic utility) and obtain some general insight into the validity of simple biologistic points of views. It remains to be seen whether some of the above mentioned aspects can add new insight.
